# Lentiviral Vectors for the Treatment and Prevention of Cystic Fibrosis Lung Disease

**DOI:** 10.3390/genes10030218

**Published:** 2019-03-14

**Authors:** Laura I. Marquez Loza, Eric C. Yuen, Paul B. McCray

**Affiliations:** 1 Stead Family Department of Pediatrics, The University of Iowa, Iowa City, IA 52242, USA; laura-marquezloza@uiowa.edu; 2 Pappajohn Biomedical Institute and the Center for Gene Therapy, The University of Iowa, Iowa City, IA 52242, USA; 3 Talee Bio, 3001 Market Street, Suite 140, Philadelphia, PA 19104, USA; eyuen@taleebio.com

**Keywords:** Cystic fibrosis, CFTR, gene therapy, safety, lentivirus, lentiviral vectors

## Abstract

Despite the continued development of cystic fibrosis transmembrane conductance regulator (CFTR) modulator drugs for the treatment of cystic fibrosis (CF), the need for mutation agnostic treatments remains. In a sub-group of CF individuals with mutations that may not respond to modulators, such as those with nonsense mutations, CFTR gene transfer to airway epithelia offers the potential for an effective treatment. Lentiviral vectors are well-suited for this purpose because they transduce nondividing cells, and provide long-term transgene expression. Studies in primary cultures of human CF airway epithelia and CF animal models demonstrate the long-term correction of CF phenotypes and low immunogenicity using lentiviral vectors. Further development of CF gene therapy requires the investigation of optimal CFTR expression in the airways. Lentiviral vectors with improved safety features have minimized insertional mutagenesis safety concerns raised in early clinical trials for severe combined immunodeficiency using γ-retroviral vectors. Recent clinical trials using improved lentiviral vectors support the feasibility and safety of lentiviral gene therapy for monogenetic diseases. While work remains to be done before CF gene therapy reaches the bedside, recent advances in lentiviral vector development reviewed here are encouraging and suggest it could be tested in clinical studies in the near future.

## 1. Introduction

Cystic fibrosis (CF) is a common autosomal recessive disease caused by mutations in the cystic fibrosis transmembrane conductance regulator (*CFTR*) gene, which encodes an anion channel. CF affects many organ systems, but the most severe symptoms arise from progressive pulmonary disease characterized by recurrent and persistent infection and inflammation, resulting in irreversible tissue remodeling that usually requires lung transplantation, or is fatal. In recent years, small molecule therapies that can partially restore CFTR function have significantly improved the outcomes for some patients [1,2,3,4,5]. However, these are life-long treatments and their benefits are mutation class specific. With over 2000 *CFTR* mutations identified to date (https://www.cftr2.org/), the need for mutation agnostic treatments remains.

Soon after *CFTR* was discovered [6], efforts to develop gene therapy for CF began. Only four years later, three patients were treated with an adenoviral vector carrying a *CFTR* expression cassette [7]. Adenoviral vectors were selected for their large carrying capacity that easily accommodates the nearly 4.5 kb *CFTR* cDNA, a promoter, and a polyadenylation signal, and they can be produced to a high titer. Subsequent studies demonstrated that adenoviral gene delivery to airway epithelia was inefficient and transient [8,9,10], which meant repeat administrations would be necessary for effective treatment. An investigation of repeated administration revealed that gene delivery efficiency was significantly reduced by host humoral and cellular immune responses [11].

Additional CF gene therapy clinical trials evaluated adeno-associated virus (AAV) [12,13] and non-viral cationic lipids complexed with plasmid DNA [14,15,16,17]. Like adenoviral vectors, both of these treatments require repeat administration due to transient transgene expression from episomes. Although multi-dose treatments were well-tolerated for both, pulmonary function improvements were modest [12,16]. Recent reviews comprehensively outline the clinical trials experience with gene therapy for CF [18,19,20].

While lentiviral vectors have not been tested in CF clinical trials, they have a sufficient packaging capacity for a *CFTR* expression cassette and can transduce non-dividing cells [21,22]. This is particularly important for CF gene therapy because most airway epithelial cells are mitotically quiescent [23,24]. Lentiviral vectors also integrate their cargo into the host genome, ensuring persistent expression for the life of the cell [25,26], which implies that if progenitor cells are transduced, daughter cells expressing the therapeutic transgene can repopulate the surface epithelium. Additionally, unlike adenoviral vectors, lentiviral vectors display low immunogenicity [27,28,29]. In a sub-group of CF individuals with more severe lung disease who may not respond to CFTR modulators, such as those with nonsense or splicing mutations, lentiviral vectors may offer particular advantages.

An important question regarding the use of lentiviral vectors for in vivo somatic cell gene therapy is safety. Because they integrate, there is a potential risk of insertional mutagenesis. Here, we will contrast features of γ-retroviral and lentiviral vector systems. We will review results from clinical trials for other diseases that raised safety issues and discuss the steps taken to address these concerns. We will also review current progress towards lentiviral gene therapy for CF disease, and other ongoing advances in the lentiviral gene therapy field.

## 2. Retroviruses

The *Retroviridae* family is composed of seven genera, including five retroviruses (α, β, γ, δ, ε), lentiviruses, and spumaviruses. This family is characterized by their diploid, single-stranded, positive sense RNA genomes, which are transcribed into viral DNA in the cytosol by the reverse transcriptase enzyme. This double-stranded DNA is then transported to the nucleus and integrates into the genome [30]. These viruses can be modified for use as replication incompetent vectors to deliver genes of interest to mammalian cells.

While several retroviruses, including α-retroviruses [31] and spumaviruses [32], have been investigated for gene therapy applications, γ-retroviruses and lentiviruses are the most extensively studied in human gene therapy clinical trials. γ-retroviruses were the first to be used in clinical trials for the treatment of a genetic disease. Some findings from these first trials raised concerns regarding the use of integrating vectors. As a consequence, many safety features were incorporated into subsequent versions of γ-retroviral and lentiviral vectors for gene therapy. Despite the excellent track record of safety and efficacy in several clinical trials, concerns regarding the safety of retroviral vectors for human gene therapy persist. For this reason, we will review the outcomes of the early γ-retroviral clinical trials and discuss the lessons from these studies that influenced the subsequent development of lentiviral vectors.

### 2.1.γ-Retroviral Gene Therapy

This retrovirus family member was the first to be used in human gene therapy clinical trials for the treatment of a genetic disorder. Severe combined immunodeficiency (SCID) comprises a group of genetic conditions that affect bone marrow-derived immune cells, resulting in impaired T and B cell function leading to severe and often lethal infections. While an HLA-matched bone marrow transplant can be curative, not all patients find a suitable match, and those that do can experience graft-vs-host disease [33]. Thus, the severity of the disease, lack of universally effective treatments, and an easily accessible progenitor cell population that could be transduced ex vivo, made SCID an ideal candidate for the development of retroviral gene therapy.

In 1990, a clinical trial for SCID due to adenosine deaminase (ADA) deficiency began, involving ex vivo delivery of the *ADA* gene to patient-derived T cells using a γ-retroviral vector [34]. In this and other pilot studies, there was evidence of partial immune reconstitution, an integrated vector, and *ADA* gene expression in the T cells that persisted, but enzyme replacement therapy was still required in all patients [34,35,36]. In this disease, genetically complemented cells have a selective advantage for survival and expansion that is inhibited by enzyme replacement therapy [36,37,38,39]. In subsequent studies with improved engraftment using nonmyeloablative conditioning, 10 patients had no deleterious effects during follow-up over a median of four years, and most did not require enzyme replacement therapy [40,41].

In 1999, clinical trials were initiated for X-linked SCID (SCID-X1) also using γ-retroviral vectors. In these studies, hematopoietic stem cells (HSC) were isolated and transduced with a γ-retroviral vector ex vivo to deliver the common cytokine receptor γ chain (γc), encoded by the *IL2RG* gene, and then returned to the patients. Twenty patients were enrolled in participating centers in France and the UK [42,43,44]. Of note, when γc expression is restored, transduced cells have a selective survival advantage [38,39,45]. Initial results were very promising, with all patients showing evidence of improved immune reconstitution soon after treatment [42,43,44]. In the years following, however, clonal T cell lymphoproliferations occurred in six of the 20 patients after γ-retroviral vector gene therapy for SCID-X1 [46,47,48,49,50]. One of these patients did not respond to leukemia treatment and eventually died. When the first case of lymphoproliferation was reported in 2002, the trials were immediately halted [51]. Trials resumed two years later, as the benefits to the treated patients without adverse effects were considered to outweigh the potential risks of clonal T cell lymphoproliferations. To further minimize the risks, the French group restricted the treatment to older children and returned fewer transduced cells to patients [52]. Ultimately, all trials were suspended a year later after the third case of clonal T cell lymphoproliferation and the death of one of the original patients were reported [53].

While all retroviruses integrate into the host genome, their integration site preferences are virus-specific [54,55,56,57]. γ-retroviral integration is enriched near enhancer and promoter regions of actively transcribed genes [55,58]. In the case of the SCID-X1 patients who experienced clonal T cell lymphoproliferation, insertions were mapped near proto-oncogenes (*LMO2*, *BMI1*, *CCND2*) [46,47,59]. These genes were dysregulated through expression driven by strong enhancer elements present within the γ-retroviral long terminal repeat (LTR), leading to lymphoproliferation. Activation insertions were also reported in people treated with γ-retroviral vectors for X-linked chronic granulomatous disease (X-CGD) and Wiskott-Aldrich Syndrome (WAS). Three people treated for X-CGD exhibited the insertional activation of genes, leading to myelodysplasia, in addition to transgene silencing by promoter methylation in two of these patients [60,61]. Similarly, seven patients treated for WAS developed acute leukemias following genotoxic insertional activations [62]. Although insertional mutagenesis is a serious adverse event, it is important to note that malignancy has not been reported in any treated ADA-SCID patients and nearly all of the treated patients from both SCID groups continued to benefit from the treatment 20 years later [63], in the face of a disease with up to 50% mortality [64,65]. The possibility exists, that there could be something unique about the pathophysiology of SCID-X1, X-CGD, and WAS that facilitated integration near oncogenes. These findings from clinical trials stimulated a number of studies to improve the safety of retroviral gene therapy vectors.

In addition to vector insertion site preference and the choice of promoters, there are other factors to consider for optimal vector design and delivery. For instance, HSC expressing exogenous multi-drug resistance 1 (*MDR1*) delivered using a retroviral vector showed a selective advantage, allowing improved expansion. When transplanted into mice, however, all animals developed a myeloproliferative disorder [66]. Separate studies demonstrated that *MDR1* or fluorescent protein gene delivery using high doses of retroviral vectors resulted in genomic instability and acquired leukemias [67]. Malignant transformation is a complex process that requires multiple aberrant processes to coincide. Retroviral insertion site preference is only one cooperating factor [48,68].

The adverse outcomes in γ-retroviral clinical trials led to the development of improved vectors developed for SCID. These improved vectors incorporated several safety features. A significant improvement was the development of a self-inactivating (SIN) γ-retroviral vector. In SIN vectors, the LTR enhancer–promoter sequences are deleted and the gene of interest is expressed from an internal promoter; strong enhancers are generally avoided. In the improved SIN γ-retroviral vector for SCID-X1, the LTR U3 enhancer from the Moloney murine leukemia virus was deleted [69]. In addition, the modified vector used the human elongation factor 1α (EF1α) promoter to drive constitutive transgene expression [69]. Cellular promoters such as EF1α have shown reduced potential to induce the expression of neighboring genes, compared to retroviral enhancer-promoters [50]. Since these modifications were introduced, there have been no reports of cancer to date in >40 treated patients [69,70,71,72,73], and Strimvelis, a γ-retroviral vector for the ex vivo treatment of ADA-SCID, was approved by the European Medicines Agency in 2016 [74]. These results indicate that stepwise vector improvements reduced the risk of insertional mutagenesis with early γ-retroviral vectors. Taken together, these studies suggest that there are at least six key factors to consider regarding retroviral vector design for gene therapy applications: (1) retroviral insertion site preference; (2) transgene promoter strength; (3) enhancer activity of the vector LTR; (4) selective survival advantage of corrected cells; (5) vector dose (vector copy number per diploid genome); and (6) predisposing factors that could lead to genotoxicity in response to the integration of an exogenous gene.

### 2.2. Lentiviral Vectors

Based on the genotoxicity associated with γ-retroviral vectors, the field has largely moved on to lentiviral vectors due to the very low to negligible genotoxicity risk. Human immunodeficiency virus (HIV) and other primate (simian (SIV)) and non-primate lentivirus species, including feline immunodeficiency virus (FIV) and equine infectious anemia virus (EIAV), are currently being assessed for their potential gene therapy applications. HIV-based lentiviral vectors differ from γ-retroviral vectors in significant ways that improve safety. First, they display an integration site preference that, while still within transcriptionally active regions of the genome, shows no preference for enhancer or promoter regions, and is therefore less likely to be genotoxic [56,57,58,75,76]. Lentiviral vector integrations map across transcribed genes, predominantly in introns. Second, since enhancer–promoter elements contribute more to genotoxicity than insertion patterns [77,78], SIN lentiviral vectors were developed to reduce genotoxicity [79,80]. Additional modifications include the use of a synthetic chromatin insulator in lentiviral vectors to reduce interactions between the inserted transgene and neighboring genes [81,82,83]. A moderate multiplicity of infection dose helps to avoid multiple integration events per cell. Ideally, an average of one integration event per cell would take place and result in monoallelic integration. Collectively, these modifications reduce the risk of genotoxicity.

Other safety features have been incorporated into lentiviral vector design and production. In addition to separating the viral genes necessary for vector production into separate plasmids, accessory genes not required for virus packaging or replication have been removed or expressed in *trans* [84,85]. For lentiviral vector production, the required components are expressed from different plasmids to reduce any possibility of recombination and production of replication competent viral particles. The vector components are usually separated into three or four plasmids: (1) *gag-pol* plasmid(s), which contain the viral structural genes and packaging signal; (2) a transgene plasmid with a heterologous promoter and gene of interest; and (3) the viral envelope glycoprotein plasmid to express the envelope and pseudotype the vector. In some cases, *rev* genes are separated from *gag-pol* as a fourth component [84,85].

## 3. Lentiviral Vector Development for Cystic Fibrosis Gene Therapy

Lentivirus vector systems have been investigated for CF applications since their development in the 1990s [22]. This includes the HIV [86,87,88,89], FIV [81,90,91,92], EIAV [29,93], and SIV [94,95,96] vector platforms. In addition to transducing non-dividing cells, another attractive feature of lentiviral vectors is the ability to alter their cell and tissue tropism by changing the envelope glycoprotein pseudotype [97]. A broad range of virus families have been tested for their tropism for airway cell types. For example, envelope glycoproteins from filoviruses [98,99], baculovirus [91,100], influenza virus [29], and Sendai virus (SeV) [27,94,96], all confer tropism to the apical surface of airway epithelia. In contrast, the widely used vesicular stomatitis virus glycoprotein (VSV-G) predominantly permits vector entry from the basolateral surface [91,101,102,103]. To enhance in vitro or in vivo delivery for VSV-G pseudotyped vectors, tight junctions can be transiently disrupted using agents such as calcium chelators [104], injury [89], or mild detergents such as lysophosphatidylcholine (LPC) [105]; however, this could increase the risk of infection in the CF lung colonized with pathogenic bacteria. Lentiviral vectors successfully delivered reporter genes to airway epithelia in well-differentiated primary culture models and animal models [22,29,81,86,87,88,90,91,94,95]. Gene expression persists for the life of the animal in mouse models [25,27,90] and can be successfully re-administered to respiratory epithelia without eliciting a blocking immune response [27,28,29].

Lentiviral vectors have also been used to deliver *CFTR* to the airways of CF animal models. HIV lentiviral vectors were successfully used to express *CFTR* to the airways of mice and rescue CF phenotypes for at least 12 months [106]. In the CF pig, which recapitulates several features of CF in humans, FIV-*CFTR* pseudotyped with the baculovirus GP64 envelope was aerosolized into the airways, and partially rescued CF phenotypes within two weeks [92]. In a step towards the validation of lentiviral gene delivery to human airways, Farrow et al. showed that the conducting airways of marmosets could be transduced with a VSV-G pseudotyped HIV-LacZ reporter vector after LPC pretreatment to disrupt tight junctions [87].

In preparation for a CF human clinical trial, Alton et al. identified an SIV-based, SeV pseudotyped SIN lentiviral vector, featuring a hybrid EF1α and cytomegalovirus (CMV) promoter. The vector was tested for efficiency in mouse and human airway epithelial cells in vitro and integration sites mapped to determine safety and vector dosing [95]. They concluded that this vector is suitable for use in humans and outlined steps towards a clinical trial in people with CF.

### 3.1. Questions Regarding the Development of Lentiviral Gene Therapy for Cystic Fibrosis

#### 3.1.1. Which Cell Types Should Be Targeted?

A major advantage of gene therapy as a treatment for CF is the potential for lasting correction of CFTR function. To accomplish this, cells with a progenitor capacity will need to be targeted. CF is primarily a disease of the airways and a focus of most gene therapy strategies is to deliver cargo to the epithelia of the proximal large airways (cartilage and submucosal gland containing; pseudostratified columnar epithelium) and distal small airways (no cartilage; simple columnar epithelium). Several progenitor cell types are distributed regionally in the conducting airways [107]. Within these regions, the progenitor cell types include basal cells (Muc5AC^−^, K5^+^, p63^+^) in the proximal cartilage containing tracheobronchial epithelium [108,109], club cells, and a population of basal and α6β4^+^ cells in the small airways [110,111]. Of note, some basal cells in the proximal airways may have cell membrane extensions that reach the airway lumen [112], while the epithelial progenitors of the small airways (basal, club, and α6β4^+^ cells) are directly accessible from the lumen [110,111]. Differentiated cells such as ciliated cells of the large and small airways are long-lived in mice (half-life of six months in the trachea and 17 months in bronchioles [113]). Robust data concerning airway cell turnover in humans are lacking. Of note, multiple studies of gene transfer to various epithelial cell types have been reported with lentiviral vectors pseudotyped with the VSV-G [87], GP64 [28], and SeV [95] envelopes. In addition, the transduction of some airway epithelial cell progenitor cell types by lentiviral vectors has been demonstrated in vitro [114] and in vivo [95].

Recent studies in mouse trachea describe a new stem cell niche that contributes to airway repair, the submucosal gland myoepithelial cells [115]. In these studies, glandular myoepithelial cells adopted a basal cell phenotype and established lasting progenitors capable of further regeneration following re-injury. Their role in human airways is not yet known. Additionally, the recent progress in single cell RNA expression profiling has also identified new airway cell types. Bulk microarray or RNA sequencing experiments demonstrate that *CFTR* is a low abundance transcript in the tracheobronchial epithelium. In two recent publications, a new cell type termed the pulmonary ionocyte, was identified using single cell RNA sequencing technology [116,117]. Ionocytes represent approximately 1% of epithelial cells in the proximal airways, but were found to express approximately 50% of all *CFTR* mRNA transcripts in the large airway epithelium of a mouse or humans [116,117]. The remaining *CFTR* mRNA transcripts were expressed at low levels in secretory and ciliated cell types [117]. These findings point out a previously unrecognized diversity of cell types and CFTR distribution in the airway epithelium [118]. While additional studies are needed to understand their place in therapeutic CF phenotypic correction, it is likely that a therapeutic benefit can be gained by correcting both long-lived terminally differentiated and progenitor cell types [119,120,121].

#### 3.1.2. What Level of Cystic Fibrosis Transmembrane Conductance Regulator Expression Must Be Achieved?

An important question for gene therapy is the level of *CFTR* expression that must be achieved in transduced cells to correct CF phenotypes. At least five studies examined the relationship between the percent of cells expressing CFTR and transepithelial Cl^−^ secretion [122,123,124,125,126]. Overall, they suggest that expressing CFTR in 10–50% of cells restores Cl^−^ secretion to wildtype levels. These studies led to the idea that correcting ~10% of the cells would restore Cl^−^ transport and correct the clinical phenotype. However, these studies do not address HCO_3_^−^ secretion or differentiate between wildtype or exogenous *CFTR* expression levels. In recent cell mixing studies using airway epithelia from CF and wildtype pigs, it was reported that as the proportion of wildtype cells increased, cAMP-stimulated Cl^−^ current increased and exhibited close to wildtype levels with a 50:50 mix of cells [127]. Interestingly, 50% wildtype cells generated only ~50% of HCO_3_^−^ current, although the amount of HCO_3_^−^ current needed to achieve clinically relevant improvement in pulmonary function is uncertain. Of note, heterozygote *CFTR^+/−^* epithelia, which produce ~50% as much CFTR as wildtype, generated ~100% of wildtype Cl^−^ current, but again, ~50% of wildtype HCO_3_^−^ current [127]. Carriers of *CFTR* mutations, however, do not commonly manifest respiratory defects, suggesting that 50% wildtype *CFTR* expression is sufficient to avoid disease and correction of ≤50% of cells may be therapeutically relevant. One interpretation of these results, combined with the recent discovery of the pulmonary ionocyte, is that it is possible that expressing greater than wildtype CFTR levels in a small number of cells might restore defects associated with CF, including Cl^−^ and HCO_3_^−^ secretion, airway surface liquid pH, and host defense abnormalities. Although achieving CFTR expression to 50% of wildtype levels should be curative (i.e., the treated CF lung would become equivalent to the healthy lung of a CF carrier), CFTR expression at as little as 10% of wildtype levels may still provide substantial improvement in pulmonary function via significant improvement in Cl^−^ current. Such an improvement would be considered a successful step in CF gene therapy on the path towards a complete cure. Additional studies are needed to better understand the therapeutic implications of these findings.

#### 3.1.3. How Can Physical Barriers to In Vivo, Somatic Cell Gene Therapy for Cystic Fibrosis Be Overcome?

The first obstacle for gene transfer to somatic cells is physical. While HSC can be readily harvested, modified ex vivo, and replaced, the modification of airway epithelial cells requires in vivo delivery. In studies of gene delivery to newborn pigs, intratracheal vector instillation using a microsprayer [128] or atomizer [92] successfully delivered the transgene to multiple regions of the airways. Similar devices could be adapted for use in humans. Alton et al. also noted that an aerosol generating nebulizer could be used for whole lung delivery in the future, but focused on testing nasal delivery devices such as catheters and a nasal spray bottle for the initial safety studies they proposed [95]. They concluded that vector passage through the devices did not compromise the transduction efficiency.

Once the vector is delivered to the desired region of the respiratory tract, the next challenge is obtaining the required transduction efficiency. Lentiviral vector transduction efficiency in the airways may be limited by several factors, including vector titers, mucociliary clearance, epithelial barrier properties, and viral envelope glycoprotein access to receptors. To overcome the rapid clearance of vectors from the airways, viscoelastic gels such as methylcellulose have been used to slow mucociliary transport and allow a more prolonged residence time of the vector on epithelial cells for increased receptor binding and entry [90,92,129,130]. While enveloped lentiviral vectors offer more production challenges than encapsidated AAV and adenovirus vectors, advances continue in this field.

#### 3.1.4. What Is the Risk-Benefit Ratio of Somatic Cell Lentiviral Gene Therapy for Cystic Fibrosis?

To date, of the more than 1350 patients treated with lentiviral vectors in clinical trials, none have evidence of insertional mutagenesis [131,132]. The majority involve the ex vivo treatment of hematopoietic stem cells for monogenetic diseases (>350 patients and 1000 patient-years, based on review of current and completed clinical trials utilizing lentiviral vectors), including SCID-X1 [133], adrenoleukodystrophy (ALD) [134], metachromatic leukodystrophy (MLD) [135], WAS [136], β-thalassemia [137], sickle cell disease [138], and others summarized in Table 1. In general, these treatments polyclonally reconstitute the hematopoietic system [133,134,135,136,137,138]. One patient in the thalassemia trial developed a clonal expansion of a population, but it spontaneously regressed without treatment [139]. In addition, in vivo somatic cell lentiviral gene therapy trials targeting the brain for MLD, X-ALD, and the retina for Stargardt’s macular degeneration and Usher syndrome type 1B are now underway. These and other in vivo clinical trials for monogenetic diseases are summarized in Table 1. Lentiviral gene therapy is also in clinical trials for non-genetic diseases such as Parkinson’s disease [140,141] and chronic HIV infection [142,143].

Among subjects receiving lentiviral gene therapy, the longest treated group are those with ALD. Within this cohort, there are patients that received treatment over 10 years ago (2–12 years), without adverse events [131,144]. In addition to the absence of adverse events, the stable engraftment of HSC was observed in nine patients at a median follow-up of three years. A notable outcome, relevant for progressive diseases such as CF, is that in eight patients treated prior to symptom onset, disease development was prevented, and three patients showed signs of re-myelination [144].

Another growing population of patients who received lentiviral therapies are those treated with US Federal Food and Drug Administration approved ex vivo chimeric antigen receptor T (CAR-T) cell therapies for malignancies [145,146]. Lentiviral vectors are the most widely used tool to deliver CAR-T treatments and we estimate that more than 1000 patients have received this therapy, with no reports of genotoxicity related to the lentiviral gene transfer [132,147,148].

Lentiviral gene therapy with HSC is the most rigorous scenario for evaluating the risk of insertional mutagenesis. We note that HSC differ in significant ways from airway epithelial cells. For example, with many diseases (e.g., SCID-X1, ADA-SCID, and X-CGD), treated HSC acquire a selective survival advantage and will expand [36,37,38,39,40,45,46,149]. Primary immunodeficiencies are also associated with an increased risk of malignancy [150,151], which may contribute to oncogenesis following gene therapy. In addition, HSC are a pluripotent, dividing cell type and are not mitotically quiescent.

Hematopoietic stem cells also express many stem cell genes, providing more transcriptionally active target genes for insertional mutagenesis. Thus, HSC transduction likely carries more risks than transducing a mitotically quiescent airway epithelial cell in the setting of CF. In addition, there is no known survival advantage for a corrected CF airway epithelial cell. We conclude that HSC are a litmus for transformation by lentiviral vectors and safety in this cell type is reassuring.

The current evidence points to a low genotoxicity risk for lentiviral vectors. Given that over 1,350 patients have been treated with lentiviral vector gene therapy to date without developing cancer, the risk of genotoxicity can be estimated to be 0–0.22% (95% confidence interval, rule of three) [152]. If patients receiving CAR-T are excluded, there are over 350 patients treated, leading to a genotoxicity risk of 0–0.86% (95% CI). As additional patients are treated with lentiviral vectors without genotoxicity, the risk will likely lessen.

How can these data be considered in the case of gene therapy for CF? An assessment of drug development candidates always depends on an analysis of the risk-benefit ratio. For the ~10% of CF individuals with nonsense mutations, splice site mutations, and other mutations for which correctors and potentiators will not be effective, one must weigh the potential benefit of effective lentiviral vector gene therapy vs. the 30–50% five-year mortality rate of lung transplantation [153] or 0–0.22% genotoxicity risk. We should also consider that life-long periodic exposure to IV vancomycin, IV aminoglycosides, IV piperacillin, or high dose ibuprofen, all have cumulative risks of serious adverse reactions. In summary, the benefit-risk analysis of lentiviral vector development supports the treatment of adults with minimal function *CFTR* mutations and advanced CF lung disease for whom no near-term options exist for CFTR-directed treatment prior to lung transplantation.

## 4. Summary

Currently, clinical trials of gene therapy with lentiviral vector systems are having a profound impact on several monogenetic diseases, including ADA-SCID, SCID-X1, ALD, MLD, X-CGD, WAS, β-thalassemia, and sickle cell disease [133,134,135,136,137,138]. The CF gene therapy field continues to make remarkable steps towards understanding barriers and developing new, more efficient gene transfer tools. While there is still progress to be made, there are many reasons to be optimistic that gene therapy for CF is on the horizon.

## Figures and Tables

**Table 1:** Current and completed gene therapy clinical trials using lentiviral vectors for monogenetic diseases.

Ex Vivo Lentiviral Gene Therapy Clinical Trials ^1^			
Disease	Autologous Cells Transplanted	ClinicalTrials.gov Identifier	Gene Delivered
β-Thalassemia Major	Genetically modified HSC	NCT01639690	*HBB*
		NCT02906202	*HBB*
		NCT03276455	*HBB*
		NCT01745120	*HBB* (LentiGlobin BB305)
		NCT02151526	*HBB* (LentiGlobin BB305)
		NCT03207009	*HBB* (LentiGlobin BB305)
		NCT02453477	*HBB* (GLOBE)
Sickle Cell Disease	Genetically modified HSC	NCT02186418	*HbF*
		NCT02247843	*βAS3-globin*
		NCT03282656	shRNA targeting *BCL11A*
		NCT02151526	*HBB* (LentiGlobin BB305)
		NCT02140554	*HBB* (LentiGlobin BB305)
ADA-SCID	Genetically modified HSC	NCT03765632	*ADA*
		NCT02999984	
		NCT01852071	
		NCT01380990	
		NTC02022696	
ART-SCID	Genetically modified HSC	NCT03538899	*DCLRE1C*
SCID-X1	Genetically modified HSC	NCT01306019	*IL2RG*
		NCT03601286	
		NCT03315078	
X-CGD	Genetically modified HSC	NCT01855685	*GP91-PHOX*
		NCT02234934	
		NCT02757911	
		NCT03645486	
WAS	Genetically modified HSC	NCT01515462	*WAS*
		NCT01347346	
		NCT01347242	
		NCT01410825	
MLD	Genetically modified HSC	NCT03392987	*ARSA*
		NCT01560182	
		NCT02559830	
ALD	Genetically modified HSC	NCT02559830	*ARSA*
		NCT01896102	*ARSA* (LentiD)
Fabry Disease	Genetically modified HSC	NCT02800070	*GLA*
Fanconi Anemia	Genetically modified HSC	NCT01331018	*FANCA*
Hemophilia A	Genetically modified HSC	NCT03818763	*F8*
Leukocyte Adhesion Defect	Genetically modified HSC	NCT03812263	*ITGB2*
Mucopolysaccharidosis Type 1, Hurler variant	Genetically modified HSC	NCT03488394	*IDUA*
Severe localized scleroderma	Genetically modified fibroblasts	NCT03740724	*MMP-1*
Epidermolysis Bullosa Dystrophica	Genetically modified fibroblasts	NCT02810951	*COL7*
**In vivo somatic cell lentiviral gene therapy clinical trials for monogenetic diseases ^2^**			
**Disease**	**Lentiviral vector injection site**	**ClinicalTrials.gov Identifier**	**Gene Delivered**
MLD	Intracerebral	NCT03725670	*ARSA*
X-ALD	Intracerebral	NCT03727555	*ABCD1*
Stargardt’s Macular Degeneration	Subretinal	NCT01367444	*ABCR* (SAR422459)
Usher Syndrome Type 1B	Subretinal	NCT01505062	*MYO7A* (SAR421869)

^1^ This table only includes gene therapy clinical trials for monogenetic diseases. There are many others using lentiviral vectors to deliver cancer treatments, such as chimeric antigen receptor T (CAR-T) therapies, not included here. ^2^ Also not included are clinical trials of intracerebral administration for sporadic Parkinson’s Disease. HSC: hematopoietic stem cells, SCID: severe combined immunodeficiency, ADA: adenosine deaminase, ART: Artemis, CGD: chronic granulomatous disease, WAS: Wiskott-Aldrich Syndrome, MLD: metachromatic leukodystrophy, ALD: adrenoleukodystrophy.
